# Alternative workflows for identifying transnational missing persons

**DOI:** 10.1016/j.fsisyn.2023.100445

**Published:** 2023-10-30

**Authors:** Molly A. Kaplan, Chloe P. McDaneld, Jessi Brown, Isabella-Marie Selden, Hua Jiang, Eugene Tan, Richard Selden, M Kate Spradley, Sara Huston

**Affiliations:** a Forensic Anthropology Center, Department of Anthropology, Texas State University, San Marcos, TX, USA; b ANDE Corporation LLC, Waltham, MA, USA; c Mary Ann & J. Milburn Smith Child Health Research, Outreach, and Advocacy Center, Ann & Robert H. Lurie Children's Hospital of Chicago, Chicago, IL, USA; d Department of Pediatrics, Northwestern University, Chicago, IL, USA

**Keywords:** Rapid DNA, DNA forensics, Missing persons investigations

## Abstract

Mass migration and migrant death at the U.S. southern border highlight the disconnectedness of the systems for transnational decedent identifications. Death investigation cases in Texas face delays and barriers at all stages of an investigation. Additionally, fragmentation of DNA databases exacerbate challenges in comparing genetic samples from unidentified human remains (UHR) and families of the missing. We sought to pilot alternative workflows for processing UHR and family reference samples (FRS) for the identification of probable migrant decedents. Primarily using Rapid DNA, but also accredited non-CODIS DNA laboratories, the piloted approaches were conducted in parallel to existing medicolegal workflows under the relevant case jurisdictional guidance. Our data show that Rapid DNA is a valid path for anthropology laboratories to support identification hypotheses and that accredited non-CODIS forensic and genetic laboratories also can support families to identify remains, especially when families reside outside of the United States.

## Highlights

• Strategies are needed to improve identification times of presumed migrant decedents.
• Using accredited, non-CODIS forensic laboratories and Rapid DNA analysis to supplement the workflow can expedite identifications.
• Rapid DNA instruments in anthropology laboratories can accelerate identifications.
• To improve UHR identifications, policies should separate medicolegal death.

## 1 Introduction

Ongoing international mass migrations highlight the disconnectedness of the systems for identifications across missing persons’ databases [1]. The situation regarding migration at the U.S.-Mexico border is particularly acute, with many political, logistical, and technological challenges that inhibit cross-border identification of missing migrants [2,3]. Cases in Texas face investigative delays and barriers at all stages from the discovery of death to DNA identifications [4]. Thousands of unidentified human remains (UHR) from presumed migrants are unidentified and hundreds more are found each year in border states [4]. Estimates on exact numbers are elusive, but current reports indicate there are nearly 8000 migrant deaths recorded in the Americas since 2014 (https://missingmigrants.iom.int/); the actual number might be much greater as remains may be located in remote areas and never recovered. In U.S. states bordering Mexico, human remains appearing to be undocumented crossers take months or years to analyze, sometimes buried without collecting biometrics [5]. Local jurisdictions are desperate for solutions to these cases. They are saddled with the costs of identifying the deceased and either storing or burying the dead.

Family members of missing migrants suffer from “ambiguous loss”—not knowing whether or not their loved one is alive [6]. Often these family members are unable or unwilling to report a missing person's case due to fear of the authorities or lacking access to transnational investigative agencies [3]. Even if a family does report a missing person's case in the United States, obtaining a match with a remains sample in the CODIS database could take years [3] or might never occur. Many unidentified cases in Texas have been notoriously buried in graves without DNA collection [3,7], a practice which appears to be ongoing, at least in some regions [8].

The DNA data from presumed migrant remains that are autopsied and DNA sampled can take years to generate. With slim resources, CODIS laboratories must prioritize forensic casework, and the DNA analyses of unidentified migrant remains typically fall far below that of sexual assault kits or homicide cases [3]. Kinship analysis of UHR occasionally requires mtDNA and Y-STR analyses in addition to autosomal STRs, but processing UHR and family reference sample (FRS) for mtDNA and Y-STRs is even more expensive and labor-intensive.

For these reasons (among others), independent efforts have developed to database STR data from remains of migrants found in Texas and Arizona along with FRS STR data [3,9,10]. These efforts have been slow. In parallel, work to build databases has taken place in countries such as Guatemala, El Salvador, and Honduras, and within each state of Mexico. Hopefully database development efforts will eventually allow connectivity across borders [10]. With separate databases developing outside of CODIS, when a family submits reference DNA samples to a single database (whether to a law enforcement unit or to a non-governmental organization, NGO), then it remains possible, if not likely, that the STR data from the family member's deceased relatives are in a different database. Furthermore, it is against U.S. regulations for non-CODIS laboratories to upload STR data to CODIS. The result is a patchwork of efforts to identify the migrant remains that is ineffective.

Texas differs from other states in its laws and jurisdictional management, with Justices of the Peace serving as the presiding authority for death investigation and only 14 medical examiners serving the state's 254 counties. In counties that lack access to forensic services, particularly in rural South Texas, UHR too often are buried without autopsy or identification efforts [11]. UHR that do receive investigative efforts face a multitude of delays in DNA processing for searches within CODIS [2,11]. Verifying hypothesized identities are delayed as well, lacking resources and practices to aid in FRS collection. DNA analysis of human remains can take 1–2 years or more to run through a forensic laboratory, depending on the quality of the specimens, available technology, and resource allocations [4].

As an initiative of the Forensic Anthropology Center at Texas State University (FACTS), Operation Identification (OpID) receives UHR referrals for cases requiring identification consultation. Specifically, OpID assists local authorities in Texas border counties with the identification of probable migrant decedents, receiving cases from both direct transfers and forensic exhumations conducted in local cemeteries. While OpID provides full service forensic anthropological analyses and case management assistance, the university laboratory does not have forensic DNA capabilities. For this reason, the FACTS laboratory is well-suited to pilot alternative workflow approaches to investigating identities of remains in Texas. The term “alternative workflow” is intended herein as a path distinct from the normal workflow for a typical CODIS laboratory-based UHR identification effort.

Rapid DNA technologies are one powerful tool emerging in recent years that could facilitate DNA analysis of FRS and UHR outside of traditional forensic DNA laboratories [12,13]. Rapid DNA can be defined as the fully-automated processing of a forensic sample to generate an STR profile, typically performed outside a conventional DNA laboratory by a non-technical operator, with results generated in approximately 2 h. Rapid DNA technologies have been in development for two decades and, as a result of the Rapid DNA Act of 2017 [14] have become an approved tool for buccal swab processing in arrestee booking stations [15,16]. For evidence processing, Rapid DNA has been validated [17] and is used in disaster victim identification [18,19], and the FBI estimates that it will be used in CODIS searches of forensic samples at police stations covered by the scope of an associated CODIS laboratory by 2025 [20]. Recently, the FBI has supported the use of Rapid DNA in DVI, providing FRS samples for Rapid DNA processing of cases in the 2023 Maui Wildfire [21]. Given that the Rapid DNA technologies are designed for non-technical users, the training for operators is minimal, making it an ideal supplemental tool for established anthropology laboratories.

Because of the long delays in UHR identifications in U.S. border counties, the study team sought to pilot alternative workflows for processing OpID UHR and corresponding FRS—primarily using Rapid DNA, but also accredited non-CODIS laboratories—for the identification of probable migrant decedents recovered in South Texas. The cases included herein were ongoing open cases, so whether to use a commercial laboratory was directed by the jurisdictional authority, not by the study team. Our role was to select cases with sufficient genetic material and hypotheses to allow for alternative testing; to supplement external laboratory testing with in-house Rapid DNA testing; and to record and describe the findings for each case. The piloted approaches were conducted in parallel to existing medicolegal workflows under the relevant case jurisdictional guidance.

## 2 Materials and methods

### 2.1 Case selection

UHR cases managed by OpID were selected for pilot alternative workflows between November 2021 through March 2023. All 21 included cases were probable migrants recovered in Texas with identity hypotheses that could be tested for family relationships. All UHR cases had sufficient genetic material to allow for supplemental testing using a non-CODIS laboratory testing route (i.e., Rapid DNA analysis or non-CODIS laboratory testing). All but two of these cases were recent referrals from counties lacking a medical examiner in which fingerprint analyses could not be completed either due to the condition of the remains or the lack of comparative antemortem records. Two older OpID cases with identity hypotheses but without genetic-based identification were also included. Identity hypotheses were generated by circumstantial evidence, such as the presence of identification cards, unique personal effects, witnessing of the death by fellow crossers, and presumptive photo identifications made by family members. For inclusion in this pilot project, all cases were managed jurisdictionally through a Justice of the Peace, the legal authority for death investigations in counties in Texas lacking a medical examiner. The authorities were responsible for provision of DNA samples from UHR to a forensic laboratory for STR analysis and CODIS upload; OpID facilitated transfer of the specimens when required. Chain of custody procedures were followed for all cases. OpID ensured that authorities made all attempts to refer family members for each pilot case to local agencies, such as sheriff's departments or consulates, for FRS collection for STR analysis and upload to CODIS. In 18 of 21 cases, the FRS donors resided outside of the United States.

### 2.2 Tracking and reporting UHR

As standard practice, for each case received, OpID conducted an initial intake in which the condition of the remains and all personal effects were documented and photographed. Based on condition, OpID also performed anthropological analyses including estimation of the biological profile (sex, age, population affinity, and stature) and assessments of trauma, pathology, and postmortem modifications. After completion of forensic anthropological examinations, all case information, including photos and descriptions of personal effects, were uploaded to the National Missing and Unidentified Persons System (NamUs). All original case numbers provided by local authorities were maintained in both internal tracking systems and on NamUs, and an OpID-specific case number was assigned upon receipt. Under the authorization of the jurisdictional authority, OpID then selected UHR samples for STR analyses, including submission for CODIS upload if not previously submitted.

### 2.3 UHR sample selection

For piloting alternative workflows, OpID selected one-to-three UHR bone samples for STR analysis by either a commercial, accredited non-CODIS forensic laboratory or in-house using the ANDE Rapid DNA Identification System. Regardless of which route, sample selection from decedent remains is affected by several factors, including interment, exhumation, environmental exposure (heat, moisture, sunlight), and wildlife scavenging. Based on the bone elements available in each case, preference was given to intact bones as they provide some natural protection against exposure and bacterial degradation. Metacarpals and metatarsals were preferred, as they have excellent DNA content, are straightforward to remove, and involve a relatively small disturbance of the remains. Additional selected samples included dense, weight-bearing postcranial elements, such as the calcaneus, talus, distal femur, or patella, and axial elements, such as the clavicle, ribs, and thoracic or lumbar vertebrae [17]. Molars and canines were also selected for cases with few postcranial elements or when UHR had evidence of scavenging and weathering. For each element selected for genetic analyses, additional codes were generated for each UHR sample and tracked in conjunction with original case codes.

### 2.4 Accredited DNA laboratory processing

All external DNA testing laboratories involved in this study (3 separate laboratories) were accredited laboratories under at least one of the major DNA testing accreditation organizations (e.g., AABB, CLIA, ASCLD, ISO 17025). Casework was conducted according to their standard operating procedures to produce STR DNA testing reports for the provided specimens.

### 2.5 Rapid DNA processing

**Fig. 1:**
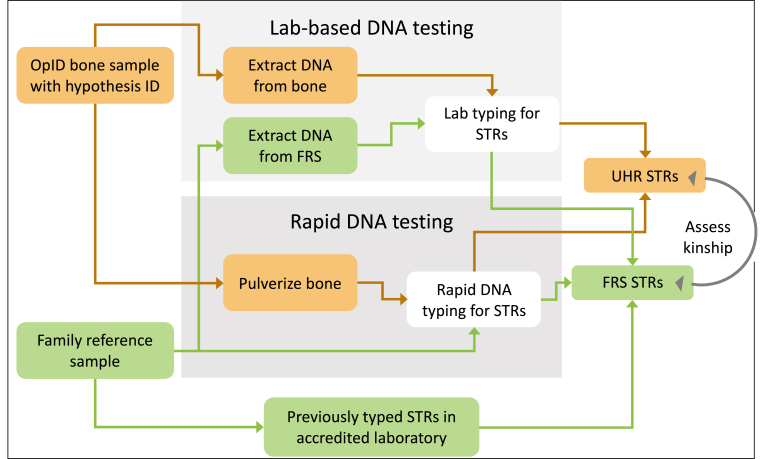
Diagram of the workflow for unidentified human remains case analyses. OpID refers to the Operation Identification initiative of the Forensic Anthropology Center at Texas State University.

The type of bone selected for analysis plays a role in DNA typing success, with denser and more compact bones typically generating better results than non-weight-bearing structures. However, any type of bone can be processed successfully, and the phalanx was preferred when available as relatively small quantities of material were required (leading to minimal disturbance of the remains). To prepare bones for Rapid DNA processing [22], they were cleaned with a soft toothbrush and running water to remove surface dirt, followed by a 30 s 10 % bleach bath to reduce surface bacteria and a thorough rinse in deionized water. Bones were then soaked in ethanol for 30 s before being allowed to dry. The bones were then wrapped in sterile Durx 770 wipe and struck with a hammer until several small pieces were generated. The small fragments were placed in a stainless steel mortar to be ground down with a pestle to fragments roughly the size of sesame seeds. 500 mg of each ground bone was added to a 2 mL tube along with 1.4 mL of ANDE® Bone Solution and 70 μL of Qiagen Proteinase K (20 mg/mL) as previously described [17]. The tubes were placed in a thermomixer at 56 °C with 500rcf agitation and incubated for 2 min (for fresh bones) to 12–18 h (for aged or degraded bones; see Ref. [19] for a description of methods for Rapid DNA processing of bones following exposure to fire). In some cases, (particularly for degraded samples), the sample liquid was concentrated with an Amicon Ultra-0.5 mL, UltraCel 10 K filter to a sample volume of 150 μL. In all cases, approximately 150 μl of the sample volume was pipetted onto an ANDE swab. A total of four swab samples were loaded into an a I-Chip for fully-automated Rapid DNA Analysis using the FlexPlex STR assay (Fig. 1).

### 2.6 Family reference samples

FRS were sought from at least two genetic relatives and collected by NGOs or appropriate consular officials for comparison. Parents or children of the missing person were always requested first, followed by full siblings and half siblings, if those were the only accessible genetic relatives. FRS were FTA blood cards or buccal swabs depending on the collecting entity. All FRS were deidentified, designated by a sample code and familial relationship. FRS were either submitted to a non-CODIS accredited DNA laboratory (n = 6) or analyzed using the ANDE Rapid DNA System (n = 14) as previously described [15,17]. In three cases, previously developed STR profiles were directly compared to UHR.

### 2.7 Kinship analyses

The FAIRS™ software package imports and decrypts run data and performs all Rapid kinship analyses. The following was calculated for each victim–reference pair.

• The number of matched loci (a matched locus was defined as one in which at least one allele is shared between the UHR and FRS profiles)
• Combined Relationship Index (CRI, the likelihood of the tested relationship for the victim–reference relative to that of two unrelated individuals). This was determined by calculating the likelihood ratio (LR) at each locus and multiplying the LR for each locus [23]. ANDE FlexPlex27 included two sets of loci that were genetically linked—loci SE33 and D6S1043; and D12S391 and vWA. The LR from one of the linked loci was included in the CRI. The kinship algorithms utilized by FAIRS met the guidelines set by the American Association of Blood Banks.

### 2.8 Reports

For non-Rapid DNA cases, the accredited genetic laboratory provided reports as per their standard operating procedures. Rapid DNA kinship reports were generated on instruments validated for non-expert use. Both the external laboratory reports and the Rapid DNA analysis kinship reports completed by the study team included kinship testing results, complete summary statistics for detected alleles, and likelihood ratios; the Rapid DNA analysis kinship reports also included probability indices for each FRS. Rapid DNA analysis kinship reports and conventional accredited laboratory kinship reports were provided to the jurisdictional authority to consider as evidence of an identification. The use of either type of DNA testing reports for confirming positive identification was at the discretion of the jurisdictional authority.

### 2.9 Descriptive analyses

Metrics were collected for each case including all relevant dates (e.g., UHR found, FRS taken, samples sent to laboratory or analyzed, date of genetic match, etc.), specimen types (i.e., bone type, saliva, blood), FRS relationships, workflow strategy, combined probability indices, and prior probabilities. Descriptive analyses were provided for length of time (in days) for various stages of analyses.

## 3 Results

From November 2021 through March 2023, 21 total UHR cases underwent alternative workflow processes for genetic testing and identification. UHR samples for 17 cases were typed using rapid DNA technology (Rapid DNA), samples for 3 cases were sent to a non-CODIS forensic DNA laboratory, and samples for 1 case was sent to a CLIA- and AABB-accredited relationship testing laboratory. FRS for 15 of the cases were typed using Rapid DNA; 3 were typed by a non-CODIS forensic laboratory, and 3 cases had DNA data (STR) provided by a forensic laboratory (1 CODIS laboratory, 2 non-CODIS forensic laboratory). As of March 2023, these alternative workflows resulted in kinship verifications for 20/21 (95.2 %) cases, with one case pending.

### 3.1 UHR sample yield

For the 21 cases, 51 UHR samples were selected for analyses, with a range of one to five samples utilized per case (Table 1). For the Rapid DNA analyses, 10 cases required a single sample-in to results-out run, and the remaining 8 cases were more significantly degraded and required a mean of 3 runs to obtain the STR data presented. In cases with multiple Rapid DNA runs, the STR results present the number of loci derived from a single sample. Details on numbers of purification, PCR, and electrophoresis repeats from the seven cases processed by the non-CODIS accredited DNA laboratory are not available. The samples that ultimately yielded STR data used for genetic comparison were: metacarpals (n = 6), teeth (n = 6), metatarsals (n = 4), ribs (n = 2), talus (n = 1), thoracic vertebra (n = 1), with one case still pending. The other 30 bone and teeth samples either were not needed for testing or yielded insufficient STR data for identity verification.

**Table 1:** Rapid DNA and non-CODIS laboratory workflow cases.

Case #	Alternative workflow approach	UHR samples	FRS sources	# Loci	Greatest Combined Probability Index (CPI)
1	Accredited forensic lab (FRS) + Rapid DNA (UHR)	vertebra, tooth, distal femur, rib	F, M	15	F: 4.12 × 10^5^
					M: 1.55 × 10^5^
2	Rapid DNA	metatarsal	C (x2)	21	C1: 8.81 × 10^8^
					C2: 7.11 × 10^10^
3	Accredited forensic lab	tooth	F, M, S	22	not provided^b^
4	Accredited forensic lab (UHR) + Rapid DNA (FRS)	vertebra	F, M, S	20, 19, 20	F: 5.33 × 10^7^
					M: 3.73 × 10^8^
					S: 9.38 × 10^9^
5	Accredited forensic lab	metacarpal	F, S (x2)	23	not provided^b^
6	Rapid DNA	talus, metatarsal	F	19	F: 5.72 × 10^9^
7	Rapid DNA	metatarsal	F, M	21	F: 1.21 × 10^8^
					M: 8.81 × 10^6^
8	Accredited genetic lab	metacarpal	F, M, S (x2)	22, 21, 31	F: 3.98 × 10^9^
					S1: 160.00
					S2: 1.41 × 10^7^
9	Accredited forensic lab (FRS) + Rapid DNA (UHR)	metacarpal	M, Mat-HS	20	M: 1.18 × 10^8^
					S: 16.33
10	Rapid DNA	tooth	S (x4)	22	cumulative: 1.98 × 10^17^
11	Rapid DNA	metacarpal	M	21	4.77 × 10^6^
12	Rapid DNA	metacarpal	M	21	1.23 × 10^8^
13	Rapid DNA	talus, metatarsal	S	20	2.07 × 10^3^^a^
14	Rapid DNA	metacarpal	F	23	4.31 × 10^8^
15	Accredited forensic lab (FRS) + Rapid DNA (UHR)	metacarpal tooth, clavicle, patella	M, C		pending
16	Rapid DNA	rib, metacarpal	S, Mat-HS (x2)	16	S: 2.15 × 10^6^
					HS1: 37.89
					HS2: 490.78
17	Rapid DNA	tooth	M	17	7.21 × 10^5^
18	Rapid DNA	calcaneus, rib, vert, tooth	F	15	3.62 × 10^4^^a^
19	Rapid DNA	calcaneus, rib, metatarsal, tooth	M, S	19, 17	M: 1.19 × 10^6^
					S: 1.91 × 10^3^
20	Rapid DNA	metatarsal	C (x3)	19	C1: 6.16 × 10^9^
					C2: 9.85 × 10^7^
					C3: 8.32 × 10^7^
21	Rapid DNA	Clavicle, tooth	M	16	5.11 × 10^5^

M = mother; F = father; S = sibling; C = child; HS = half-sibling.

The prior probability for all Rapid DNA cases is 50.0 %.

a CPI normally would be too low for a cold hit identity; however, since these cases had other circumstantial evidence, the low CPI was sufficient to support the hypothesis.

b The CPI was not provided by the external laboratories; however, the likelihood ratios for relatedness were >1.8 quadrillion (Case #3) and >6.7 billion (Case #5).

### 3.2 FRS obtained

STR data from 43 total FRS were used in the alternative workflows process, and all were compared to UHR. FRS from 37 relatives were tested, including 6 blood FTA cards and 31 buccal swabs; STR data from a prior laboratory was provided from 6 FRS. Between 1 and 4 relatives contributed FRS for each case (Table 1). Despite requesting multiple family members, 7 cases had only one parent available, and 1 case had only a genetic full sibling. Relatives included mothers (n = 12), fathers (n = 9), children (n = 6), full siblings (n = 13), and half-siblings (n = 3) (Table 1).

### 3.3 Likelihood ratios

Among the Rapid DNA analysis kinship reports, combined parentage indices ranged from 36,245 to over 6 billion, with a mean of over 1 billion and a median of 117,761,997 (Table 1).

### 3.4 Time required to complete the UHR identification by an alternative workflow

**Fig. 2:**
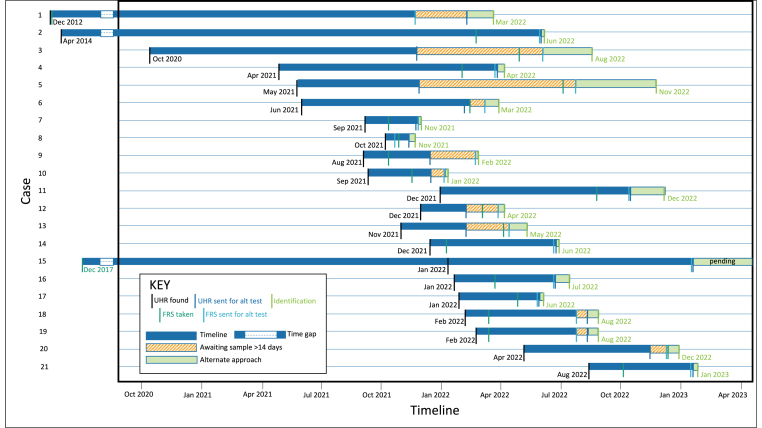
Timelines for cases piloted with alternative workflow strategies. The blue bars indicate the time that the case was investigated prior to an alternate workflow; the orange bars indicate the time from when an alternate workflow strategy commenced to when both UHR and FRS samples were available for testing; the green bars indicate the time to carry out the alternate workflow DNA testing.

For the 21 cases that underwent alternative workflows, the median number of days from when UHR were found until an identification was verified was 193.5 days, with a range of 50 to 3378 days (Fig. 2). In all cases, the alternative workflow expedited the identification. The time for alternative workflow identification was calculated as the number of days from when the first sample was obtained by the genetic laboratory until the date a genetic match report was produced. The median number of days for alternative workflow identifications was 34.5 days, with a range of 3–262 days (Fig. 2). The cases involving Rapid DNA were resolved in a median of 28.5 days.

### 3.5 Time required to complete the UHR identification by Rapid DNA processing

The mean labor expended per Rapid DNA case was 2 h including 15 min to procure samples, 15 min of hands-on time (exclusive of incubation time), 30 min of data analysis and Quality Control, and 60 min of report writing and quality review.

## 4 Discussion

For more than a decade, border jurisdictions in the United States have struggled to manage the identifications of UHR from presumed migrant deaths. We have been involved with research on exploring ways to improve stakeholder communication and DNA data sharing [2,24] and suggestions for improvements have been rare [10]. The ongoing delays in returning human remains are exacerbated by the increase in border deaths in recent years [8]. The flow of migrants into the United States is not expected to recede, so border regions must be equipped to manage the increase in deaths. This is an ongoing crisis that cannot be met with existing infrastructure.

The scientific tools used in the cases presented here are not new, but their application to the identification of missing migrants and the management of DNA data represents a dramatically new approach. To abide by U.S. state and federal laws that forbid non-CODIS laboratories from accessing DNA databases, we were only able to apply the alternative workflow strategies to cases with a working identity hypothesis for which we could process, test, and compare both UHR and FRS in one-to-one comparisons, rather than search large databases.

In Texas, there is a shortage of medicolegal experts to manage the overwhelming caseload of migrant deaths that are not suspected to be due to foul play. Other states have varying degrees of access to forensic identity services through local medical examiners and cooperation with non-profit groups that can assist with identity investigations. CODIS laboratories in Texas and across the United States are capable of handling the identifications of UHR but are backlogged with forensic casework such that the identification of migrant remains is typically a low priority. Many of the cases in this study were good candidates for an alternative workflow since the families were outside of the United States and did not have clear paths for providing FRS to CODIS laboratories. A feasibility study could help assess to what extent alternative workflows as described here would be able to address the totality of the thousands of cases pending identifications in border regions. With remains stored at a large number of sites, a regional approach to Rapid DNA implementation may be reasonable.

As of March 2023, these alternative workflows resulted in kinship verifications for 20/21 (95.2 %) of the cases attempted. These cases might have been resolved through the traditional CODIS laboratory mode, but the timeline of such prior cases led us to pilot alternative workflows rather than risk taking the typical years to identify the decedents. The oldest case (#2) took over nine years to resolve from the time the UHR was found, with the UHR found in 2012, the FRS taken 10 days later. This extended timeline is not unusual, in that FRS often are in databases separate from UHR given the split silos of DNA databases. Once an ID hypothesis was generated in June 2021 from non-DNA data, the case could be worked via an alternate workflow rather than awaiting the backlogged testing of a forensic laboratory. The alternate workflow (Rapid DNA for this case) then resolved the case in 4 months.

The quickest case (#8) to resolve took 50 days from when UHR were found in early October 2021, FRS provided later that month, and then an identification confirmed in late November 2021. When considering only the 18 resolved recent transfer cases (excluding the two long-term cases), the longest case took 1.8 years and the median time to identification was 190.5 days from when the remains were found.

To date (and to our knowledge), 18/21 cases have resulted in return of the decedents to the families. In several cases, families were aware of the recovery of their possible loved one from communication with local law enforcement or border patrol agents and were anxious to receive a positive identification to be able to mourn the deceased. The alternative workflows allowed families to receive the remains of their loved ones in a timelier manner than conventional processing in CODIS laboratories. One of the external laboratory cases was paid for out-of-pocket by the family at their request. This case, at a commercial accredited non-CODIS genetic laboratory, resulted in a kinship match in 27 days from first sample arrival, however, this independent funding for identity testing undermines the equity we strive for in serving decedents. The Rapid DNA cases were all processed without charge on a humanitarian basis.

Rapid DNA was successful in supporting the identity hypotheses in all 18 cases for which it was applied. A randomized study would need to compare the utility of Rapid DNA in comparison to traditional and non-traditional workflows. Our findings merely indicate the potential of these alternatives. In the two low CPI cases, because the Rapid DNA technology was being used to support an existing hypothesis, the low CPI was sufficient to reinforce the strong circumstantial evidence for positive identification. For future cases where Rapid DNA data might be compared to a kinship database, policies for the threshold of the CPI as indicative of a non-exclusion should be considered, as should standard training and validation processes for non-expert use in an anthropology laboratory. DNA processing is often criticized for its cost. However, the cost of UHR storage for months or years, body transport for medical examination, and exhumations if remains are buried prior to identification, far exceed the costs of DNA analyses. Our data support the placement of Rapid DNA instruments in investigative agencies—in this case anthropology laboratories—to assist their identification efforts.

## 5 Conclusion

Accounting for migrant deaths at the U.S.-Mexico border is a significant current humanitarian challenge. Our data show that Rapid DNA analysis is one valid path to support identification hypotheses and that accredited but non-CODIS laboratories also can support families to identify remains. The substantial improvement in identifications for these cases demand a consideration for implementing Rapid DNA routinely into UHR casework to expedite identifications. Comparing Rapid DNA data from UHR to FRS in CODIS or other missing persons databases would extend the power of the technology to identify more UHR cases. Embracing alternative workflows to identifications that are most expeditious and reliable, then systematizing these processes through improved practices, will serve to enhance identification efforts, especially for under-resourced border counties, overloaded medical examiners struggling with identifications, and back-logged DNA laboratories. Policy strategies to expand the utility of such alternative workflows should include separating the identity process of UHR from the cause and manner of death investigations that require additional medicolegal procedures.

## Author contributions

Conceptualization: MKS, SH, RS; Case management: MAK, CPM, MKS; Funding support: RS; Alternative workflow testing: JB, IMS, HJ, ET, RS; Data curation and analyses: MAK, SH; Manuscript preparation: MAK, SH; Manuscript editing: MKS, RS.

## Declaration of competing interest

Jessi Brown, Eugene Tan and Richard Selden are employees of and Isabella-Marie Selden was a student intern at ANDE Corporation.
